# Erratum: An exactly solvable, spatial model of mutation accumulation in cancer

**DOI:** 10.1038/srep46900

**Published:** 2017-10-20

**Authors:** Chay Paterson, Martin A. Nowak, Bartlomiej Waclaw

Scientific Reports 6: Article number: 39511; 10.1038/srep39511 published online: 12 22 2016; updated: 10 20 2017.

A Supplementary Information data file and Supplementary Mathematica notebook file were omitted from the original version of this Article. This has been corrected in the PDF and HTML version of the Article.

